# Supplementary material for: A Critical View of the Application of the APEX Software (Aqueous Photochemistry of Environmentally-Occurring Xenobiotics) to Predict Photoreaction Kinetics in Surface Freshwaters
Source: Molecules. 2019 Dec 18;25(1):9. doi: 10.3390/molecules25010009 (PMC7017383; doi:10.3390/molecules25010009)
Supplement: Supplementary file 1 [file molecules-25-00009-s001.zip › 3.2.4_gcc-4.4.0.zip!/3.2.4_gcc-4.4.0/3.2.4_gcc-4.4.0/doc/octave/html/interpreter/Finding-Roots.html]

Finding Roots - Untitled


Next: Products of Polynomials,
Previous: Evaluating Polynomials,
Up: Polynomial Manipulations


---

### 27.2 Finding Roots

Octave can find the roots of a given polynomial. This is done by computing
the companion matrix of the polynomial (see the `compan` function
for a definition), and then finding its eigenvalues.

— Function File: **roots** (v)  
> For a vector v with N components, return
> the roots of the polynomial
>
> ```
>           v(1) * z^(N-1) + ... + v(N-1) * z + v(N)
> ```
>
> As an example, the following code finds the roots of the quadratic
> polynomial
>
> ```
>           p(x) = x^2 - 5.
> ```
>
> ```
>           c = [1, 0, -5];
>           roots(c)
>           =>  2.2361
>           => -2.2361
> ```
>
> Note that the true result is
> +/- sqrt(5)
> which is roughly
> +/- 2.2361.
>
>
>
> **See also:** compan.

— Function File: **compan** (c)  
> Compute the companion matrix corresponding to polynomial coefficient
> vector c.
>
> The companion matrix is
>
> ```
>                _                                                        _
>               |  -c(2)/c(1)   -c(3)/c(1)  ...  -c(N)/c(1)  -c(N+1)/c(1)  |
>               |       1            0      ...       0             0      |
>               |       0            1      ...       0             0      |
>           A = |       .            .   .            .             .      |
>               |       .            .       .        .             .      |
>               |       .            .           .    .             .      |
>               |_      0            0      ...       1             0     _|
> ```
>
> The eigenvalues of the companion matrix are equal to the roots of the
> polynomial.
>
>
>
> **See also:** poly, roots, residue, conv, deconv, polyval, polyderiv, polyinteg.

— Function File: [multp, indx] = **mpoles** (p)  
— Function File: [multp, indx] = **mpoles** (p, tol)  
— Function File: [multp, indx] = **mpoles** (p, tol, reorder)  
> Identify unique poles in p and associates their multiplicity,
> ordering them from largest to smallest.
>
> If the relative difference of the poles is less than tol, then
> they are considered to be multiples. The default value for tol
> is 0.001.
>
> If the optional parameter reorder is zero, poles are not sorted.
>
> The value multp is a vector specifying the multiplicity of the
> poles. multp(:) refers to multiplicity of p(indx(:)).
>
> For example,
>
> ```
>           p = [2 3 1 1 2];
>           [m, n] = mpoles(p);
>             => m = [1; 1; 2; 1; 2]
>             => n = [2; 5; 1; 4; 3]
>             => p(n) = [3, 2, 2, 1, 1]
> ```
>
>
>
>
> **See also:** poly, roots, conv, deconv, polyval, polyderiv, polyinteg, residue.
